# The cadenza woodwind dataset: Synthesised quartets for music information retrieval and machine learning

**DOI:** 10.1016/j.dib.2024.111199

**Published:** 2024-12-04

**Authors:** Gerardo Roa Dabike, Trevor J. Cox, Alex J. Miller, Bruno M. Fazenda, Simone Graetzer, Rebecca R. Vos, Michael A. Akeroyd, Jennifer Firth, William M. Whitmer, Scott Bannister, Alinka Greasley, Jon P. Barker

**Affiliations:** a Acoustics Research Centre, University of Salford, UK; b Hearing Sciences, Mental Health and Clinical Neurosciences, School of Medicine, University of Nottingham, UK; c School of Music, University of Leeds, UK; d Department of Computer Science, University of Sheffield, UK

**Keywords:** MIR, Audio, Ensemble, Deep learning

## Abstract

This paper presents the Cadenza Woodwind Dataset. This publicly available data is synthesised audio for woodwind quartets including renderings of each instrument in isolation. The data was created to be used as training data within Cadenza's second open machine learning challenge (CAD2) for the task on rebalancing classical music ensembles. The dataset is also intended for developing other music information retrieval (MIR) algorithms using machine learning. It was created because of the lack of large-scale datasets of classical woodwind music with separate audio for each instrument and permissive license for reuse. Music scores were selected from the OpenScore String Quartet corpus. These were rendered for two woodwind ensembles of (i) flute, oboe, clarinet and bassoon; and (ii) flute, oboe, alto saxophone and bassoon. This was done by a professional music producer using industry-standard software. Virtual instruments were used to create the audio for each instrument using software that interpreted expression markings in the score. Convolution reverberation was used to simulate a performance space and the ensembles mixed. The dataset consists of the audio and associated metadata.

Specifications Table

**Table:** 

Subject	Data Science: Applied Machine Learning
Specific subject area	Developing algorithms for Music Information Retrieval (MIR), music signal processing and deep learning*.*
Type of data	Digital audio files Metadata in *.json format
Data collection	Nineteen scores were randomly selected from the OpenScore String Quartet corpus. The synthesis of these as woodwind ensembles was performed by a sound engineering professional using professional software. The scores were loaded into the music notation software Steinberg's Dorico. The string parts were allocated to flute, oboe, clarinet (or alto saxophone) and bassoon. Virtual instruments were used to create the audio for each instrument: Miroslav Philharmonik 2 by IK Multimedia for the saxophone and Intimate Studio Winds by 8Dio for the other parts. The quartets were then imported into Avid's Pro Tools where they were mixed and reverberation added. Metadata were generated.*.*
Data source location	Institution: University of Salford City/Town/Region: Salford Country: UK*.*
Data accessibility	Repository name: Zenodo Data identification number: 10.5281/zenodo.12664932 Direct URL to data: https://zenodo.org/records/12664932**[**1**]** Dataset freely available and has a Creative Commons Attribution-Share Alike 4.0 International License
Related research article	N/A*.*

## 1 Value of the Data

• Music Information Retrieval (MIR) is a field of research concerned with using computational methods to extract information from, analyze and understand music. Nowadays, the dominant method for developing MIR tools is machine learning using deep neural networks (DNNs). The Cadenza Woodwind dataset might be used to develop algorithms for MIR tasks such as: demixing (music source separation), transcription and instrument activity detection.
• Researchers require large datasets with several hours of audio to develop music processing algorithms using modern machine learning methods. For some music algorithm development, researchers need access to the mixed ensemble and also each instrument isolated as separate audio. While there are several public datasets of non-classical music in this form, the only public large-scale classical music data lacks enough hours of woodwind instruments. Cadenza Woodwind was developed to fill this gap.
• Ideally, a dataset would be drawn from live recordings of musicians playing, but no such data exists because the number of hours required for machine learning makes this difficult and expensive to collect. Consequently, Cadenza Woodwind uses professional tools to synthesize musical scores. Researchers using the dataset will need to ensure their machine learning algorithms can generalize to real recordings.

## 2 Background

The Cadenza project aims to improve the processing of music for those with a hearing loss through open machine learning challenges. Listening to music can benefit health and well-being [2], but it can be a poorer experience for those with a hearing impairment. Quieter passages of music might be inaudible, pitch perception can be poorer, and identifying and distinguishing lyrics and instruments can be harder [[3], [4], [5]]. The World Health Organisation estimates that, by 2050, 2.5 billion people will have some form of hearing loss, with at least 700 million of those requiring treatment [6]. The main treatment for hearing loss is hearing aids, but the efficacy of these for music is mixed [[7], [8], [9], [10]].

One of the Cadenza machine learning challenge tasks is the rebalancing of classical music ensembles. To enable this challenge, a new training dataset of woodwind ensembles was developed.

Modern professional sound engineering tools feature high quality virtual instruments and reverberation algorithms based on measurements of real instruments and spaces. Increasing amounts of music for TV, film and radio are created using such techniques. These also enabled the dataset to be generated..

## 3 Data Description

The data is available in two files, (i) the metadata in JSON format (CadenzaWoodwind.json) and, (ii) the audio files in a compressed package (CadenzaWoodwind.zip). The audio is in 16-bit FLAC format with a 44,100 Hz sampling frequency. The dataset includes 38 ensembles organized in separate folders:

1. 19 folders for bassoon, clarinet, flute, and oboe quartets with folder names matching their OpenScore identifiers (e.g. sq10490761).
2. 19 folders for bassoon, alto saxophone, flute and oboe quartets with folder names using the OpenScore identifiers and a “_2” suffix (e.g. sq10490761_2). Only the alto saxophone and the corresponding mixture are stored in the “_2” folders, as the bassoon, flute, and oboe parts are the same as (1).

The metadata file contains details for each ensemble (see Table 1), including duration, relative paths to isolated stems of each instrument and mixture tracks for the whole quartet, and the train/validation split for CAD2. For ensembles featuring bassoon, clarinet, flute, and oboe, the item keys and track names match their identifiers from the OpenScore dataset.

**Table 1:** Description of JSON metadata.

Field name	Description	Example
Dataset	Name of the dataset.	“CadenzaWoodwind”
Name	The OpenScore identifier	“sq10490761”
Duration	The duration of the audio file in seconds	“1778.7125″
Instruments	List of instruments in the mixture	[“Oboe”, “Bassoon”, “Clarinet”, “Flute”]
stems_path	List with the relative path to each isolated instrument in the same order as “Instruments” field	[“sq10490761/Oboe.flac”, “sq10490761/Bassoon.flac”, “sq10490761/Clarinet.flac”, “sq10490761/Flute.flac”]
mixture_path	Relative path to the audio mixture	“sq10490761/mix_ sq10490761.flac”
Set	Dataset split assigned for CAD2 challenge	“train”

Ensembles that use alto saxophone instead of clarinet are differentiated by a "_2″ suffix in the track name. Table 2 illustrates the differences in the metadata between ensembles using a clarinet and an alto saxophone. These differences are reflected in four fields: name, instruments, and paths to isolated instruments and mixture.

**Table 2:** Differences in metadata for items with and without the “_2″ suffix.

Field name	Without suffix	With suffix
Name	“sq10490761”	“sq10490761_2″
Instruments	[“Oboe”, “Bassoon”, “Clarinet”, “Flute”]	[“Oboe”, “Bassoon”, “Sax”, “Flute”]
stems_path	[“sq10490761/Oboe.flac”, “sq10490761/Bassoon.flac”, “sq10490761/Clarinet.flac”, “sq10490761/Flute.flac”]	[“sq10490761/Oboe.flac”, “sq10490761/Bassoon.flac”, “sq10490761_2/Sax.flac”, “sq10490761/Flute.flac”]
mixture_path	“sq10490761/mix_ sq10490761.flac”	
	“sq10490761_2/mix_ sq10490761_2.flac”	

## 4 Experimental Design, Materials and Methods

### 4.1 Score selection

The OpenScore String Quartet corpus was used for the music scores [11]. The corpus was chosen because it was large enough to create the audio duration required and was available in the public domain with an appropriate open license to allow reuse. There are over 100 scores from 40 composers of historic string quartets, all created by volunteers. In music, it is quite common for historic works with one orchestration to be repurposed for a different group of instruments. Consequently, these string quartets were repurposed as woodwind quartets. For a performance, some manual reorchestration would be done to allow for the different capability of the instruments, but there was not the resource to reorchestrate 6 h of music per instrument – see *Rendering each instrument* for the consequences of this.

The scores to be selected were chosen randomly. Because the synthesis of the instruments involved manual work, the random selection was biased towards choosing longer scores to reduce time required for the rendering. This bias was introduced by weighting the random process that selected the scores by the duration of each score. The list of selected scores was then filtered so there were not more than two pieces by any composer. The aim was to have at least 6 h of woodwind ensembles, and this led to 19 scores being selected. The scores were in the form of musicXML files, an open file format that encodes Western musical notation.

### 4.2 Synthesis

During the rendering of the sound, sound engineering decisions had to be made such as the choice of the virtual instruments, reverberation and mixing. This was done by a professional music producer with guidance to make the music as high quality and as natural sounding as possible. But the process needed to be as automated as possible, because there was a limited amount of time that the music producer could spend on the task.

### 4.3 Rendering each instrument

Two orchestrations for each score were to be created: (i) flute, oboe, clarinet and bassoon; and (ii) flute, oboe, alto saxophone and bassoon. This required five individual instrument tracks to be made per score. Virtual instruments were used to create the audio for each instrument: Miroslav Philharmonik 2 by IK Multimedia for the saxophone and Intimate Studio Winds by 8Dio for the other parts. The music producer auditioned a variety of virtual instruments and picked these two based on what sounded authentic, with good tonality and timbre. A further consideration was which virtual instrument could perform the necessary articulations without requiring excessive manual intervention.

Music notation software, Steinberg's Dorico, was used to synthesize each instrument. This was done because Dorico could interpret the expressive markings of the music scores in the musicXML files to create more naturalistic sounding audio. Within Dorico, key-switches were assigned by the music producer to change the articulation of the virtual instrument based on the expression text found in the score. One example of this would be that any short notes in the score triggered a sound from the staccato algorithm rather than the generic legato patch.

The virtual instrument used was comprised of audio samples recorded of live woodwind musicians playing their instruments. The result of this is that the synthesis cannot make a sound that the actual instrument itself cannot make. The scores were written originally for string quartet. Normally when reorchestrating music for new instruments, playability would be considered. But this was not necessary because virtual instruments were being used. However, there were some brief sections of music where the written score for a string instrument was out of range of the woodwind instrument. For these passages, the woodwind instrument did not sound. There was an issue that some expression markings were not applicable to woodwind players. Tremolo was particularly problematic and sounded very artificial when played by the virtual instrument. After consultation with professional saxophonist, a technique known as flutter tongue was used instead of tremolo. This resulted in a much more realistic sound.

The audio was exported for each instrument from Dorico at 48 kHz 24 bit as Broadcast Wave Format (BWF) files.

### 4.4 Adding reverberation and mixing

The audio was imported into Avid's Pro Tools. Reverberation was added using a convolution reverb, using an impulse response from the Royal Tropical Institute, Amsterdam, in Avid's Space Impulse Response Library. The room reverberation was used on each individual instrument to create an ambience around the instrument and place it further back on the soundstage, to resemble a more natural capture. The dry audio from Dorico was also blended with the reverberated version to manipulate the sense of how far back in the room the player was. This level was different based on the timbre of the instrument and the sound of the room it was placed in. The level of dry signal blended against the reverberated signal (at 0 dBFS) was: −12.4; −10.6; −9.8; −9.8, and −17.3 dBFS for the flute, oboe, clarinet, saxophone and bassoon respectively. The level of the bassoon was lower than the other instruments as it contained significantly more low frequency energy than the other instruments. This prevented the sound being boomy, unbalanced and unnatural.

The audio was exported from Pro Tools at 48 kHz, 24bit. The individual stems and mixture were treated in the same way so that the positional information of the individual instruments on the soundstage was the same for the isolated stems and the ensemble.

## Limitations

The limitations of the data already discussed above centre around the use of string quartet scores and the rendering of these using woodwind virtual instruments. It is worth noting that when most machine learners use these music databases, they will take liberties with the rendering to improve generalisation of their algorithms. For example, common data augmentation methods will remix different parts of the score with no regard for the original orchestration or timing [12]. A final limitation is that the diversity of the scores is restricted, with only 19 historic examples being used. Notwithstanding, the publication of Cadenza Woodwind significantly increases the diversity of classical music and orchestrations available at a scale useful for deep learning.

## Ethics Statement

The authors have read and followed the ethical requirements for publication in Data in Brief. The work reported in this paper does not involve human subjects, animal experiments, or any data collected from social media platforms.

## CRedit Author Statement

**Gerardo Roa Dabike**: Conceptualization, Methodology, Data Curation, Writing - Original Draft, Writing - Review & Editing; **Trevor J. Cox**: Conceptualization, Methodology, Software, Data Curation, Writing - Original Draft, Writing - Review & Editing, Supervision, Funding acquisition; **Alex Miller**: Investigation, Methodology, Resources, Writing - Original Draft; **Bruno Fazenda**: Conceptualization, Funding acquisition; **Simone Graetzer**: Conceptualization, Writing - Review & Editing, Funding acquisition; **Rebecca R. Vos**: Conceptualization; **Michael A. Akeroyd**: Conceptualization, Funding acquisition; **Jennifer Firth**: Conceptualization; **William M. Whitmer**: Conceptualization, Funding acquisition, **Scott Bannister:** Conceptualization; **Alinka Greasley**: Conceptualization, Funding acquisition; **Jon P. Barker**: Conceptualization, Funding acquisition.

## Data Availability

ZenodoCadenza Challenge (CAD2): databases for rebalancing classical music task (Original data).
